# Case Report: Prenatal Genetic Counseling to Parents of Fetuses Suspected of Having Ambiguous Genitalia

**DOI:** 10.3389/fped.2020.569548

**Published:** 2021-01-13

**Authors:** Takeshi Sato, Tomohiro Ishii, Yu Yamaguchi, Yosuke Ichihashi, Daigo Ochiai, Hiroshi Asanuma, Tatsuo Kuroda, Tomonobu Hasegawa

**Affiliations:** ^1^Department of Pediatrics, Keio University School of Medicine, Tokyo, Japan; ^2^The center for Differences of Sex Development, Keio University Hospital, Tokyo, Japan; ^3^Department of Clinical Genetics, Gunma Children's Medical Center, Gunma, Japan; ^4^Department of Obstetrics and Gynecology, Keio University School of Medicine, Tokyo, Japan; ^5^Department of Urology, Keio University School of Medicine, Tokyo, Japan; ^6^Department of Pediatric Surgery, Keio University School of Medicine, Tokyo, Japan

**Keywords:** ambiguous genitalia, case report, differences in sex development, prenatal genetic counseling, sex assignment

## Abstract

The occurrence of fetuses suspected of having ambiguous genitalia will likely increase in the future. Currently, the impact of prenatal genetic counseling on parents' understanding and psychological preparedness has not been addressed. We provided prenatal genetic counseling to parents of two fetuses suspected of ambiguous genitalia. Case 1: At 22 weeks of gestation, swelling of the labia majora, and a clitoris-like structure were noted despite 46,XY detected in amniotic fluid cells. Case 2: At 28 weeks of gestation, bladder exstrophy and a scrotum-like structure were noted. At 32 weeks (Case 1) and 37 weeks (Case 2) of gestation, we shared information with parents regarding the possible difficulty of legal sex assignment at birth, and a scenario for registration of the birth certificate. At birth, both babies presented with ambiguous genitalia. For both cases, the parents remained calm on seeing their baby's genitalia for the first time. After a month, we shared medical information with parents, including karyotype, testosterone production capacity, and surgical schedule. In both cases parents assigned their respective baby's legal sex as male. Several months later, parents were questioned on prenatal genetic counseling. Case 1: Mother, “I was prepared to address our baby's genitalia calmly.” Father, “I understood the procedure of legal sex assignment.” Case 2: Mother, “Without counseling, I would have been more upset and worried.” Father, “We were assured that multidisciplinary experts would support us.” Prenatal genetic counseling provides reassurance to parents, who remain informed and emotionally secure throughout the legal sex assignment of their child.

## Introduction

Recent advances in imaging devices, including ultrasonography (US) and magnetic resonance imaging, allow operators to observe the external genitalia of the fetus in detail (1). Because the occurrence of fetuses suspected of having ambiguous genitalia is expected to increase in the future, prenatal genetic counseling for ambiguous genitalia is recommended (2). Currently, there have been no reports focusing on the impact of prenatal genetic counseling on parents not expecting the birth of a fetus with ambiguous genitalia.

Here, we report the results of prenatal genetic counseling for parents of two fetuses suspected of having ambiguous genitalia and assessed the impact of counseling on the parents' understanding and psychological preparedness.

## Clinical Reports

The institutional review board of the Keio University School of Medicine approved the study (No. 20150104 and 20170130). We obtained informed consent from the parents for inclusion in this study.

Timelines of Case 1 and 2 are shown in Tables 1, 2, respectively.

**Table 1:** Timeline of Case 1.

**Gestational and chronological age of the baby**	**Response from the parents**	**Results of examinations on the baby**	**Interventions**
22 weeks of gestation		Intra-uterine growth restriction, persistent left superior vena cava and female-like external genitalia on ultrasonography 46,XY karyotype at amniocentesis	
32 weeks of gestation	The parents fully understood the postnatal procedures of legal sex assignment.		The DSD medical team informed parents that: (i) legal sex assignment may be difficult at birth; (ii) genetic and endocrinological examinations and imaging studies requiring several weeks may be needed to collect medical information to help determine the legal sex; and (iii) birth registration could be delayed for medical reasons.
Birth (34 weeks of gestation)	The parents appeared to be unaffected psychologically on looking at the baby's ambiguous genitalia.	Birth weight, 1,262 g (−3.2 SD) A phallus length of 15 mm, a common urogenital sinus at perineum, and bilateral gonads at the labioscrotal swelling Persistent left superior vena cava and patent ductus arteriosus on cardiac ultrasonography	Emergent cesarean section
18 days of life			The patent ductus arteriosus was closed surgically.
24 days of life	The parents themselves assigned and serenely registered their baby's sex as male.	46,XY karyotype of the umbilical cord blood Testes in both sides of labioscrotal swelling No uterus Serum testosterone and anti-Müllerian hormone levels consistent with males Appropriate surgical procedures	The DSD medical team shared medical information of the baby with the parents for sex assignment.
4 months of age	The psychological statuses of both parents were assessed as stable.		

*DSD, differences in sex development*.

**Table 2:** Timeline of Case 2.

**Gestational and chronological age of the baby**	**Response from the parents**	**Results of examinations on the baby**	**Interventions**
28 weeks of gestation		Omphalocele, bladder exstrophy, spinal deformity, and ambiguous genitalia with a scrotum-like structure and no apparent penis on ultrasonography	
37 weeks of gestation	The parents fully understood the postnatal procedures of legal sex assignment.		The DSD medical team informed the parents that (i) legal sex assignment may be difficult at birth; (ii) that genetic and endocrinological examinations and imaging studies over 2–4 weeks may be needed to collect medical information to help determination of legal sex; and (iii) birth registration can be delayed legally for medical reasons.
Birth (38 weeks of gestation)	The parents appeared to be unaffected psychologically on looking at the baby's ambiguous genitalia.	Birth weight, 2,366 g (−1.3 SD) No apparent phallus and meatus The scrotum-like labioscrotal folds on the perineum and the gonads in the inguinal canals Imperforate anus, omphalocele, bladder exstrophy, and spinal deformity	Elective cesarean section
1 day of life			Surgery for omphalocele and colostomy was performed.
17 days of life	The parents themselves assigned and serenely registered their baby's sex as male.	46,XY karyotype of peripheral blood Testes in the inguinal canals No uterus Substantial testosterone production at human chorionic gonadotropin loading test and levels of serum anti-Müllerian hormone consistent with males The difficulty of surgical procedures Presumed male identity	The DSD medical team shared medical information of the baby with the parents for sex assignment.
6 months of age	The psychological statuses of both parents were assessed as stable.		

### Case 1

A mother, aged 29 years with a history of one gravidity and no parity, presented for consultation. Her clinical course was uneventful. At 22 weeks of gestation, intra-uterine growth restriction and persistent left superior vena cava were detected by routine US. Although the fetus presented with female-like external genitalia, prenatal amniocentesis testing showed 46,XY karyotype. This implied that the fetus had differences in sex development (DSD).

**Figure 1:**
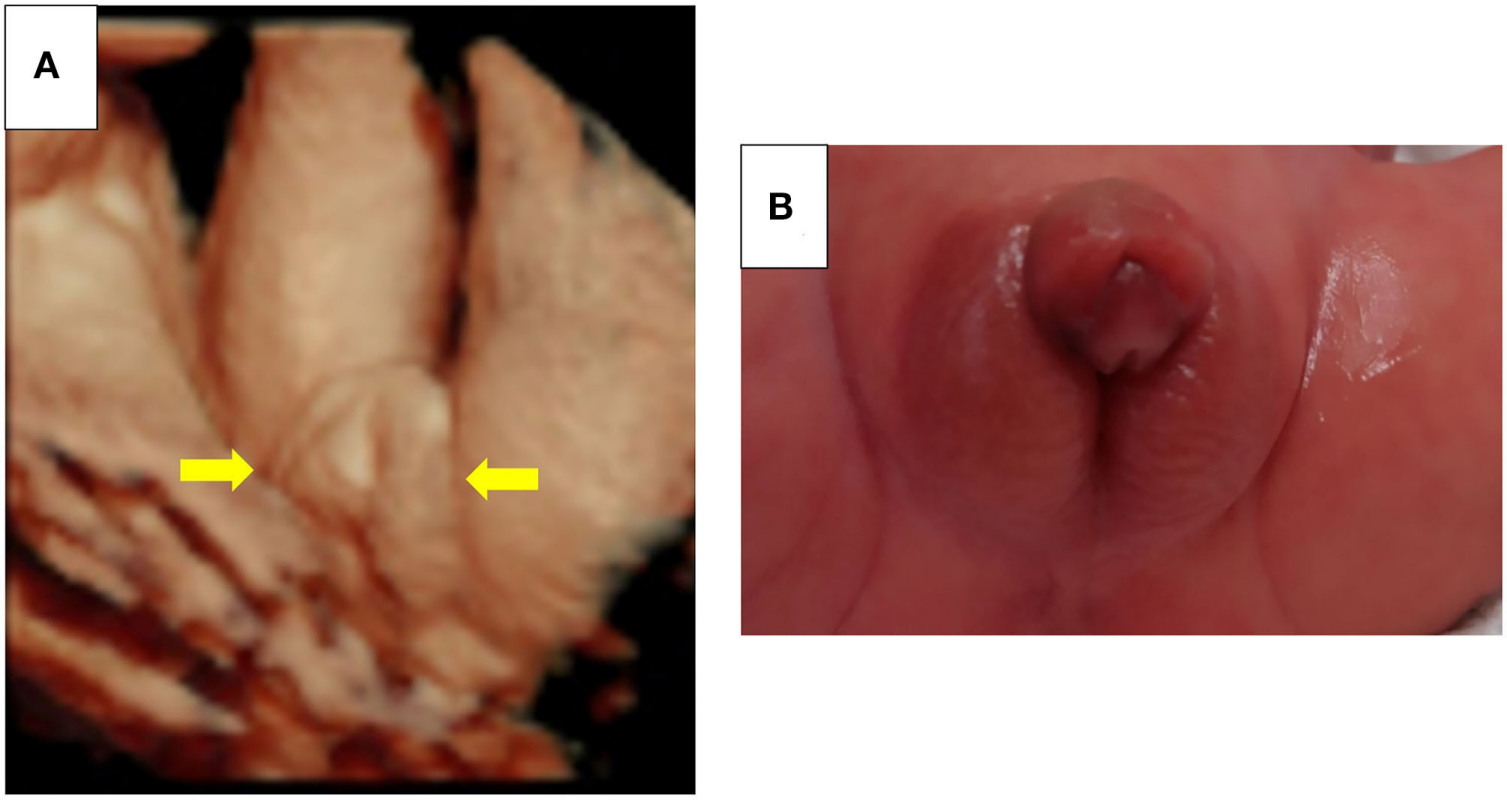
**(A)** Fetal ultrasonography in Case 1. Labia majora-like and clitoris-like structures were observed (yellow arrows). **(B)** Appearance of the external genitalia in Case 1. Physical examination of external genitalia showed a phallus length of 15 mm and a common urogenital sinus at the perineum. Permission to use the photo was granted by the parents.

At 31 weeks of gestation, she was referred to our hospital because of potential difficulties of legal sex assignment. Three-dimensional US showed ambiguous genitalia with labia majora-like and clitoris-like structures (Figure 1A). At 32 weeks of gestation, the professional DSD medical team informed parents that: (i) legal sex assignment may be difficult at birth, (ii) genetic and endocrinological examinations and imaging studies over the course of several weeks may be needed to collect medical information to help determine the legal sex, and (iii) birth registration could be delayed for medical reasons. Parents fully understood the postnatal procedures of legal sex assignment and decided to postpone the assignment until results of thorough examinations were obtained after birth.

At 34 weeks of gestation, an emergency cesarean section was performed due to pregnancy-induced hypertension. The baby's birth weight was 1,262 g (−3.2 SD). Physical examinations on external genitalia showed a phallus length of 15 mm, a common urogenital sinus at the perineum, and bilateral gonads at the labioscrotal swelling (Figure 1B). The parents appeared to be unaffected psychologically on looking at the baby's ambiguous genitalia at birth, according to the attending physicians. As scheduled, legal sex assignment was postponed. Genetic and endocrinological examinations and abdominal US were initiated. Cardiac US showed persistent left superior vena cava and patent ductus arteriosus. At 18 days of age, the patent ductus arteriosus was closed surgically. At 24 days of age, the DSD medical team shared with the parents the following medical information: (i) 46,XY karyotype of the umbilical cord blood; (ii) testes on both sides of the labioscrotal swelling; (iii) no uterus; (iv) serum testosterone (1.01 ng/mL, on day 11) and anti-Müllerian hormone levels (72.4 ng/mL, on day 0) consistent with males; and (v) appropriate surgical procedures. Finally, the parents themselves assigned and calmly registered their baby's sex as male.

When the baby was 4 months old, the attending physicians assessed the general condition of the baby and the mother, as well as the psychological statuses of both parents, as stable. On interviewing the parents, his mother said, “I was nervous when I searched ‘ambiguous genitalia' on the Internet. However, after counseling, I was able to prepare myself to address the issue of our baby's genitalia calmly.” The infant's father indicated, “Before birth, I did not fully understand the consequences of ambiguous genitalia, but I understood the procedure of legal sex assignment. After counseling, I realized that the professional team helped us.”

### Case 2

**Figure 2:**
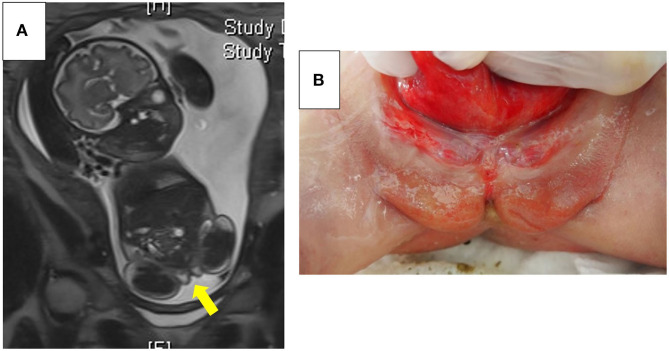
**(A)** Fetal magnetic resonance imaging in Case 2. A scrotum-like structure was noted (a yellow arrow). **(B)** Appearance of the external genitalia in Case 2. No apparent phallus or urethral meatus at the perineum was observed. Scrotum-like labioscrotal folds were noted. Permission to use the photo was granted by the parents.

A mother with a history of one gravidity and no parity aged 38 years old presented with an uneventful clinical course. At 28 weeks of gestation, omphalocele, bladder exstrophy, and spinal deformity were suspected by routine US. The fetus appeared to have ambiguous genitalia with a scrotum-like structure and no apparent penis (Figure 2A).

At 36 weeks of gestation, she was referred to our hospital for management of complications and legal sex assignment. At 37 weeks of gestation, the DSD medical team informed the parents that: (i) legal sex assignment may be difficult at birth, (ii) genetic and endocrinological examinations and imaging studies over 2–4 weeks may be needed to collect medical information to help in determination of legal sex, and (iii) birth registration can be delayed legally for medical reasons. The parents understood the procedures and decided to postpone legal sex assignment until results of thorough examinations could be obtained after birth.

At 38 weeks of gestation, an elective cesarean section was performed. The baby's birth weight was 2,366 g (−1.3 SD). An imperforate anus, in addition to omphalocele and bladder exstrophy, and spinal deformity were noted, suggesting the diagnosis of an omphalocele-exstrophy-imperforate anus-spinal defects, also defined as an OEIS complex. Physical examinations on the external genitalia showed no apparent phallus and meatus, but revealed the scrotum-like labioscrotal folds on the perineum and the gonads in the inguinal canals (Figure 2B). Parents appeared calm on observing the baby's abdomen and external genitalia, according to the attending nurses. As previously decided, the assignment of legal sex was postponed. Genetic and endocrinological examinations and abdominal US were initiated. At 1 day old, surgery for omphalocele and colostomy was performed. At 17 days old, the DSD medical team shared the following medical information: (i) 46,XY of peripheral blood, (ii) testes in the inguinal canals, (iii) no uterus, (iv) substantial testosterone production at human chorionic gonadotropin loading test (basal 0.6 ng/mL on day 7, peak 3.73 ng/mL on day 10) and levels of serum anti-Müllerian hormone (46.3 ng/mL, on day 0) consistent with males, (v) the difficulty of surgical procedures, and (vi) that male identity was presumed based on the previous literature (3). Finally, the parents themselves assigned and calmly registered their baby's sex as male.

When the baby was 6 months old, the attending physicians assessed the general condition of the baby and the mother, as well as the psychological statuses of both parents, as stable. In a follow-up interview, his mother indicated, “When I saw him at birth, I was upset to some extent, although I had received genetic counseling. Without counseling, I would have been more upset and worried.” His father reported, “We were assured that multidisciplinary experts supported us. Of course, we were worried, but we trusted you.”

## Discussion

We report the outcome of prenatal genetic counseling to parents of two cases of fetuses suspected of having ambiguous genitalia, and several months after birth, parents were interviewed with regard to the psychological impact of the child's condition. In both cases, the parents appeared calm, from the moment they first looked at their baby's genitalia at birth up to when they assigned and registered their baby's legal sex as male. Based on the parents' comments, we considered that the prenatal genetic counseling: (i) provided relief to parents, (ii) helped parents to prepare themselves to look at their baby's genitalia, and (iii) helped parents to fully understand the procedures involved in assignment of legal sex.

Prenatal genetic counseling for ambiguous genitalia leads to better understanding of the condition by the parents, compared to postnatal genetic counseling only. Thanks to prenatal genetic counseling, parents could calmly, and productively discuss the child's condition with the clinical staff. Moreover, with sufficient time until delivery parents could research “ambiguous genitalia” and ask questions. Currently, during postnatal genetic counseling, we rush the parents' understanding to obtain consent in a short time, so as to initiate examination for legal sex assignment as soon as possible. This has been problematic, as mothers often became upset, were tired, or may not have fully understood the clinical condition. From the parent's standpoint, when the fetus had other complications, prenatal genetic counseling would have more advantages. Our two fetuses needed to be evaluated soon after birth owing to suspicion of vascular anomalies in Case 1 and gastrointestinal anomalies in Case 2. If genetic counseling for sex assignment were provided after birth, parents would not have the time to fully understand.

Prenatal genetic counseling has a potential risk of increasing the anxiety of parents (4). To minimize anxiety in parents, we should decide cautiously in each case what information to share with parents during genetic counseling and when to provide genetic counseling, considering the mother's educational, and psychological statuses. If the mother's condition is compromised, our first priority should be to emphasize that ambiguous genitalia in itself is not a fatal disease and can be treated surgically, and that the professional DSD medical team will support parents. We consider pediatricians, who are familiar with endocrinology, genetics, and total and through-life management of DSD, to be the most eligible individuals to perform prenatal genetic counseling for the following reasons: (i) examinations for sex assignment require specialized knowledge in endocrinology and genetics; (ii) growth and development of the child, as well as related complications, must be observed carefully; (iii) from adolescence, puberty induction and assisted reproductive medicine may be needed; and (iv) parents are concerned about a variety of issues, such as medical expense and recurrence risk in the next child, and require continuous emotional support. Currently, the appropriate time in which to provide prenatal genetic counseling is unknown. In our opinion, because emergency cesarean section is sometimes needed, initial genetic counseling should occur soon after ambiguous genitalia is suspected, and repeated genetic counseling is desirable to verify the parents' understanding at 34–36 weeks of gestation. Generally, the father, who is more familiar with the mother's character than anyone else and is concerned about her emotional condition, plays the most important role in helping her to cope with her anxiety. Sometimes, it may be important to ask the father's opinion and wishes before counseling the mother. Both fathers of the fetuses described herein were able to support the mother emotionally. If the partner is absent or unsupportive, we should closely cooperate with obstetricians, and pay special attention to the mother's emotional status.

The limitations of this study included selection bias and descriptive evaluation. We selected patients, provided prenatal genetic counseling, and evaluated the effect of the counseling arbitrarily. To plan prospective studies in the future, the selection criteria for patients, indications for prenatal genetic counseling, and quantitative methods of evaluating the counseling should be determined prudently to avoid unexpected anxiety in parents, especially pregnant, or puerperal women. We believe that it is appropriate to determine selection criteria, method of providing counseling, and quantitative methods of evaluation with caution in current clinical practice, similar to the practice where this study was conducted. Interviews several months after delivery may have been less disturbing to parents, but had the following limitations: (i) parents' recall might be incomplete or they might have forgotten problems, and (ii) parents might hesitate to express negative comments in front of the interviewers. In addition, the two fetuses with systemic complications were not considered representative of fetuses with ambiguous genitalia. A larger number of fetuses and parents is needed to evaluate the impact of prenatal genetic counseling accurately.

The above-mentioned benefits and limitations of prenatal genetic counseling for ambiguous genitalia have also been applied to other fetuses with potential difficulties in legal sex assignment, for example, fetuses proven to have the 45,X/46,XY mosaicism (5). Considering that the ambiguous genitalia observed in our two cases were first identified by the obstetricians' US, we should emphasize the significance of prenatal genetic counseling to obstetricians, and encourage them to quickly refer such mothers to hospitals where the professional DSD medical team is based.

In summary, we reported the impact of prenatal genetic counseling to parents of two fetuses suspected of ambiguous genitalia. Prenatal genetic counseling provides reassurance to parents, and parents remain informed and emotionally stable throughout the assignment of legal sex to their child.

## Data Availability Statement

The raw data supporting the conclusions of this article will be made available by the authors, without undue reservation.

## Ethics Statement

The studies involving human participants were reviewed and approved by the institutional review board of the Keio University School of Medicine. Written informed consent to participate in this study was provided by the participants' legal guardian/next of kin. Written informed consent was obtained from the minor(s)' legal guardian/next of kin for the publication of any potentially identifiable images or data included in this article.

## Author Contributions

TS, TI, YI, and TH conceptualized and designed the study, collected data, carried out the analyses, drafted the initial manuscript, reviewed, and revised the manuscript. YY, DO, HA, and TK designed the data collection, carried out the analyses, and critically reviewed the manuscript.

## Conflict of Interest

The authors declare that the research was conducted in the absence of any commercial or financial relationships that could be construed as a potential conflict of interest.
